# Supplementary material for: Serum Asprosin Correlates with Indirect Insulin Resistance Indices
Source: Biomedicines. 2023 May 28;11(6):1568. doi: 10.3390/biomedicines11061568 (PMC10295799; doi:10.3390/biomedicines11061568)
Supplement: Supplementary file 1 [file biomedicines-11-01568-s001.zip › biomedicines-2372604-supplementary.pdf]

Number	Gender	Age (years)	Asprosin (ng/ml])	Fasting plasma glucose (mmol/l)	Fasting plasma glucose (mg/dL)	TCHOL (mmol/L)	LDL (mmol/L)	HDL (mmol/L)	HDL (mg/dL)	TG (mmol/L)	TG (mg/dL)	Body height (cm)	Waist circumference (cm)	Neck circumference (cm)	Body mass (kg)	BMI	WHtR	NHtR	TyG	TG/HDLc	TyG-BMI	TyG-WC	TyG-NC	TyG-WHtR	TyG-NHtR	Obesity (1-yes, 0-no)	Hba1c (%)
1.	F	73	113,44	4,2	75,6	4,33	2,57	1,31	50,6577	1,14	100,9698	167	112	37	85,7	30,72896	0,670659	0,221557	8,247131	0,870229	253,4258	923,6786	305,1438	5,53101	1,827209	1	
2.	F	60	32,13	5,3	95,4	4,62	2,94	0,96	37,1232	2,77	245,3389	164	125	41	80,7	30,00446	0,762195	0,25	9,367572	2,885417	281,069	1170,946	384,0704	7,139918	2,341893	1	4,6
3.	M	31	81,85	5,6	100,8	4,68	3,5	0,9	34,803	2,47	218,7679	182	155	48	162,9	49,17884	0,851648	0,263736	9,308003	2,744444	457,7568	1442,74	446,7841	7,927145	2,454858	1	
4.	F	55	101,33	4,9	88,2	3,66	2,54	0,78	30,1626	1,88	166,5116	163	113	41	92,2	34,7021	0,693252	0,251534	8,901525	2,410256	308,9016	1005,872	364,9625	6,170996	2,239034	1	5,8
5.	F	44	138,39	5,2	93,6	6,61	4,94	1,28	49,4976	1,22	108,0554	163	120	41	100,5	37,82604	0,736196	0,251534	8,528527	0,953125	322,6004	1023,423	349,6696	6,27867	2,145212	1	
6.	M	34	46,18	5,7	102,6	5,46	3,93	1,08	41,7636	2,51	222,3107	175	129	45	128,5	41,95918	0,737143	0,257143	9,341767	2,324074	391,9729	1205,088	420,3795	6,886217	2,402169	1	
7.	F	48	53,52	4,9	88,2	3,93	2,99	1,07	41,3769	0,97	85,9129	169	111	40	104,9	36,72841	0,656805	0,236686	8,239794	0,906542	302,6345	914,6171	329,5918	5,411936	1,950247	1	5,6
8.	F	54	5,91	5,6	100,8	2,54	1,16	0,83	32,0961	1,47	130,1979	160	73	32,5	55,8	21,79688	0,45625	0,203125	8,789047	1,771084	191,5738	641,6004	285,644	4,010003	1,785275	0	5,5
9.	M	51	67,26	5,2	93,6	5,3	4,15	0,96	37,1232	1,7	150,569	177	153	49	149,1	47,59169	0,864407	0,276836	8,860305	1,770833	421,6769	1355,627	434,1549	7,658907	2,452853	1	5,8
10.	M	30	43,57	4,5	81	3,54	2,18	0,95	36,7365	1,44	127,5408	186	143	41	150,8	43,58885	0,768817	0,22043	8,549738	1,515789	372,6733	1222,613	350,5393	6,573186	1,88462	1	5
11.	M	37	488,37	5,2	93,6	5,17	3,4	1,03	39,8	2,93	259,12	174	170	57	211,8	69,9564	0,977011	0,327586	9,403174	2,84466	657,8122	1598,54	535,9809	9,18701	3,08035	1	5,7
12.	M	45	52,28	6,4	115,2	4,63	3,8	1,14	44,0838	1,28	113,3696	187	162	48,5	168,2	48,09975	0,86631	0,259358	8,784176	1,122807	422,5166	1423,036	426,0325	7,609821	2,278249	1	5,9
13.	M	43	20,96	5,1	91,8	5,97	4,61	1,11	42,9237	2,43	215,2251	184	95	39	87,9	25,9629	0,516304	0,211957	9,19815	2,189189	238,8107	873,8242	358,7278	4,749045	1,949608	0	5,1
14.	M	30	6,11	4,9	88,2	5,59	4,11	1,42	54,9114	1,31	116,0267	171	100	40	80	27,35885	0,584795	0,233918	8,54028	0,922535	233,6522	854,028	341,6112	4,994316	1,997726	0	5,4
15.	M	23	20,48	5,2	93,6	4,14	3,32	0,91	35,1897	1,5	132,855	188	107	41	104,7	29,62313	0,569149	0,218085	8,735142	1,648352	258,7623	934,6601	358,1408	4,971596	1,905004	0	4,9
16.	F	59	71,47	5,9	106,2	6,95	3,15	1,25	48,3375	5,11	452,5927	164	99	40	87,7	32,60708	0,603659	0,243902	10,08717	4,088	328,9131	998,6298	403,4868	6,089206	2,460285	1	6
17.	M	40	55,54	5,7	102,6	4,5	2,9	1,27	49,1109	0,78	69,0846	170	136	44	129,3	44,74048	0,8	0,258824	8,173023	0,614173	365,665	1111,531	359,613	6,538418	2,115371	1	5,7
18.	F	32	274,68	5,2	93,6	4,27	2,71	1,03	39,8301	2,24	198,3968	167	130	44	175,2	62,82047	0,778443	0,263473	9,136152	2,174757	573,9374	1187,7	401,9907	7,111975	2,40713	1	5,9
19.	F	57	69,2	5,4	97,2	3,78	1,75	1,65	63,8055	0,86	76,1702	167	97	38	97,2	34,85245	0,580838	0,227545	8,216594	0,521212	286,3684	797,0096	312,2306	4,772513	1,869644	1	
20.	F	41	57,85	4,9	88,2	4,12	1,87	2,1	81,207	0,93	82,3701	170	89	31	74,1	25,64014	0,523529	0,182353	8,197682	0,442857	210,1897	729,5937	254,1282	4,291728	1,494871	0	5,7
21.	F	75	13,84	4,4	79,2	5,31	3,34	1,48	57,2316	1,31	116,0267	164	79	31	53,3	19,81707	0,481707	0,189024	8,432649	0,885135	167,1104	666,1793	261,4121	4,062069	1,593976	0	6,5
22.	F	41	81,54	5,4	97,2	4,13	2,78	0,92	35,5764	1,86	164,7402	171	89	37	76	25,9909	0,520468	0,216374	8,987993	2,021739	233,6061	799,9314	332,5557	4,677961	1,94477	0	5,4
23.	M	45	136,69	5,4	97,2	4,87	3,5	0,84	32,4828	2,26	200,1682	178	152	57	163,9	51,72958	0,853933	0,320225	9,182782	2,690476	475,0214	1395,783	523,4185	7,841476	2,940554	1	5,8
24.	M	64	20,48	5,4	97,2	2,57	1,26	0,75	29,0025	3,04	269,2528	176	112	39	88,7	28,63507	0,636364	0,221591	9,479274	4,053333	271,4397	1061,674	369,6917	6,032265	2,100521	0	6,2
25.	F	35	15,84	4,9	88,2	4,15	2,8	1,09	42,1503	1,22	108,0554	165	88	30	78,7	28,90725	0,533333	0,181818	8,469104	1,119266	244,8185	745,2811	254,0731	4,516855	1,539837	0	5
26.	F	45	129,91	6	108	6,23	4,59	1,61	62,2587	1,89	167,3973	163	110	38	102,2	38,46588	0,674847	0,233129	9,109354	1,173913	350,3993	1002,029	346,1555	6,147417	2,123653	1	5,5
27.	M	43	19,26	5,6	100,8	6,45	2,22	1,22	47,1774	5,56	492,4492	187	108	42	105,5	30,16958	0,57754	0,224599	10,11938	4,557377	305,2975	1092,893	425,0141	5,844349	2,272802	1	5
28.	F	54	80,93	11,1	199,8	4,54	2,93	1,26	48,7242	1,67	147,9119	167	108	34	103,4	37,07555	0,646707	0,203593	9,600787	1,325397	355,9544	1036,885	326,4267	6,208892	1,954651	1	7,2
29.	M	40	11,86	6,6	118,8	4,07	2,77	0,85	32,8695	2,86	253,3102	182	114	43	114,6	34,59727	0,626374	0,236264	9,618909	3,364706	332,788	1096,556	413,6131	6,025031	2,272599	1	5,4
30.	F	48	48,28	6	108	5,32	3,87	1,28	49,4976	1,44	127,5408	168	132	43	144,5	51,19756	0,785714	0,255952	8,83742	1,125	452,4544	1166,539	380,0091	6,943687	2,261959	1	5,7
31.	M	31	38,6	6,1	109,8	4,46	3,49	1,11	42,9237	0,97	85,9129	182	139	45	146,2	44,13718	0,763736	0,247253	8,458847	0,873874	373,3497	1175,78	380,6481	6,460328	2,091473	1	5,6
32.	F	51	88,35	5,5	99	5,08	3,7	1,21	46,7907	1,97	174,4829	174	123	35	99,7	32,93037	0,706897	0,201149	9,063799	1,628099	298,4743	1114,847	317,233	6,407169	1,823178	1	5,5

33.	M	45	49,92	6,4	115,2	4,75	2,93	1,42	54,9114	0,89	78,8273	187	157	48	160	45,75481	0,839572	0,256684	8,420782	0,626761	385,2913	1322,063	404,1975	7,069854	2,161484	1	5,7
34.	M	31	3,61	4,9	88,2	3,83	2,3	1,46	56,4582	0,76	67,3132	171	91	38	75,3	25,75151	0,532164	0,222222	7,995816	0,520548	205,9044	727,6193	303,841	4,255083	1,776848	0	5,4
35.	F	67	30,94	5,611111	101	6,15468	2,3274	2,92218	113,0007	1,97575	174,9922	168	81	34	69	24,44728	0,482143	0,202381	9,086715	0,676122	222,1454	736,0239	308,9483	4,381095	1,838978	0	
36.	F	74	48,52	4,722222	85	3,56868	1,21542	1,78434	69,00043	1,29835	114,9949	162	96	39	79	30,10212	0,592593	0,240741	8,494392	0,727636	255,6992	815,4616	331,2813	5,033713	2,044946	1	
37.	F	66	47,37	4,333333	78	6,465	3,64626	1,75848	68,00042	2,29187	202,9909	155	97	40	82	34,13111	0,625806	0,258065	8,976723	1,303324	306,3855	870,7421	359,0689	5,617691	2,316574	1	
38.	M	61	11,19	6,611111	119	5,76678	3,05148	0,93096	36,00022	3,84989	340,9848	170	120	41	105	36,33218	0,705882	0,241176	9,917814	4,135398	360,3358	1190,138	406,6304	7,00081	2,391943	1	
39.	F	43	3,71	5,555556	100	4,18932	1,9395	1,83606	71,00044	0,93707	82,99629	167	68	30	51,5	18,46606	0,407186	0,179641	8,330819	0,51037	153,8374	566,4957	249,9246	3,39219	1,496554	0	
40.	F	66	29,58	4,777778	86	7,00806	3,7497	1,9395	75,00047	2,90153	256,9885	164	91	32	72,85	27,08581	0,554878	0,195122	9,310231	1,49602	252,1752	847,2311	297,9274	5,166043	1,816631	0	
41.	M	60	9,29	4,444444	80	3,98244	2,22396	1,39644	54,00033	0,83546	73,99669	164	110	42	85	31,60321	0,670732	0,256098	7,9929	0,598278	252,6013	879,219	335,7018	5,361091	2,046962	1	
42.	F	77	21,29	5,166667	93	5,09442	2,74116	1,73262	67,00042	1,33222	117,9947	154	90	34	72	30,35925	0,584416	0,220779	8,610092	0,768905	261,396	774,9083	292,7431	5,031872	1,900929	1	
43.	F	49	21,58	4,777778	86	7,55112	4,49964	1,75848	68,00042	2,8225	249,9888	152	104,5	37	74,4	32,20222	0,6875	0,243421	9,282616	1,605079	298,9208	970,0334	343,4568	6,381799	2,259584	1	
44.	F	67	140,13	5,111111	92	7,83558	4,93926	1,57746	61,00038	2,87895	254,9886	167	98	35,5	91,2	32,70106	0,586826	0,212575	9,36986	1,825054	306,4044	918,2463	332,63	5,498481	1,991797	1	
45.	F	35	11,28	4,222222	76	5,01684	2,09466	2,30154	89,00055	1,32093	116,9948	163	75,5	31,5	60	22,58271	0,46319	0,193252	8,399715	0,573933	189,6883	634,1785	264,591	3,890666	1,623258	0	
46.	F	60	11,39	3,555556	64	8,17176	5,14614	1,39644	54,00033	3,54506	313,986	160	78	32	59	23,04688	0,4875	0,2	9,215084	2,538641	212,3789	718,7766	294,8827	4,492354	1,843017	0	
47.	M	39	6,73	5,5	99	5,09442	2,53428	1,26714	49,0003	2,78863	246,989	187	109	45	116,1	33,20084	0,582888	0,240642	9,411316	2,200728	312,4636	1025,833	423,5092	5,485741	2,264755	1	
48.	F	60	12,61	4,277778	77	4,9134	2,99976	1,62918	63,00039	0,62095	54,99754	169	79	33	66	23,10843	0,467456	0,195266	7,657947	0,381143	176,9632	604,9778	252,7122	3,57975	1,495339	0	
49.	F	67	30,81	4,833333	87	4,9134	1,91364	1,83606	71,00044	2,52896	223,99	154	89,5	32	75,3	31,75072	0,581169	0,207792	9,184362	1,377384	291,6101	822,0004	293,8996	5,337665	1,908439	1	
50.	F	77	41,76	4,5	81	5,32716	2,61186	1,99122	77,00048	1,55802	137,9938	150	95	33	71,4	31,73333	0,633333	0,22	8,628511	0,782445	273,8114	819,7085	284,7409	5,464724	1,898272	1	
51.	F	78	15,93	6,388889	115	5,87022	3,20664	1,39644	54,00033	2,74347	242,9891	158	103,5	33,5	78,7	31,5254	0,655063	0,212025	9,544802	1,964617	300,9037	987,887	319,7509	6,252449	2,02374	1	
52.	M	51	9,54	4,666667	84	4,9134	2,24982	1,49988	58,00036	2,51767	222,99	182	109	38	92	27,77442	0,598901	0,208791	9,144797	1,678581	253,9915	996,7828	347,5023	5,476829	1,909353	0	
53.	M	63	9,83	4,611111	83	3,56868	1,21542	1,78434	69,00043	1,29835	114,9949	180	106,5	40	94,1	29,04321	0,591667	0,222222	8,470581	0,727636	246,0129	902,1169	338,8232	5,01176	1,882351	0	
54.	F	62	24,69	6,166667	111	4,73238	2,09466	2,30154	89,00055	0,71127	62,99718	163	93	37	73,7	27,73909	0,570552	0,226994	8,159473	0,309041	226,3364	758,831	301,9005	4,655405	1,85215	0	
55.	F	32	19,89	5,388889	97	3,77556	1,34472	1,39644	54,00033	2,24671	198,9911	169	84	34	69,5	24,33388	0,497041	0,201183	9,174824	1,608884	223,2591	770,6852	311,944	4,560268	1,845823	0	
56.	M	61	9,43	8,5	153	5,5599	2,89	1,08612	42,00026	6,22079	550,9754	181	98	39	95	28,99789	0,541436	0,21547	10,64898	5,727535	308,798	1043,6	415,3103	5,765747	2,294532	0	
57.	F	60	33,77	5,333333	96	7,31838	4,47378	1,49988	58,00036	2,9354	259,9884	149	87,5	32,5	63,9	28,78249	0,587248	0,218121	9,431838	1,95709	271,4718	825,2858	306,5347	5,538831	2,05728	0	
58.	M	78	25,12	7,222222	130	4,96512	2,14638	1,70676	66,00041	2,44993	216,9903	164,5	107	40,5	92,6	34,21994	0,650456	0,246201	9,55424	1,435427	326,9455	1022,304	386,9467	6,214612	2,35226	1	
59.	M	64	26,77	6,722222	121	2,99976	1,34472	1,11198	43,00027	1,18545	104,9953	171,5	120	49	104,2	35,42742	0,699708	0,285714	8,756559	1,066071	310,2223	1050,787	429,0714	6,127038	2,501874	1	
60.	F	65	46,61	5,444444	98	4,5255	0	1,08612	42,00026	4,34665	384,9828	152	126	44	105	45,44668	0,828947	0,289474	9,845019	4,001998	447,4234	1240,472	433,1808	8,161003	2,849874	1	
61.	F	73	77,17	7,444444	134	5,3013	0	1,70676	66,00041	5,06921	448,9799	152,5	90	36	75,05	32,27089	0,590164	0,236066	10,31167	2,970078	332,7664	928,0504	371,2201	6,085576	2,43423	1	
62.	F	62	10,91	4,722222	85	5,8185	2,89632	2,48256	96,0006	0,94836	83,99625	149,5	85	33	57,2	25,59255	0,568562	0,220736	8,180276	0,382009	209,3542	695,3235	269,9491	4,650993	1,80568	0	
63.	F	68	84,35	4,722222	85	4,49964	1,4223	1,44816	56,00035	3,55635	314,9859	162,5	106	37	83	31,43195	0,652308	0,227692	9,502032	2,455771	298,6674	1007,215	351,5752	6,198249	2,16354	1	
64.	M	65	9,5	4,833333	87	5,58576	3,1032	1,52574	59,00037	2,06607	182,9918	164	96	40	70,6	26,24926	0,585366	0,243902	8,982202	1,354143	235,7761	862,2914	359,2881	5,257875	2,190781	0	
65.	F	62	50,78	5,611111	101	6,05124	3,72384	1,4223	55,00034	1,96446	173,9922	160	99	35	75	29,29688	0,61875	0,21875	9,080984	1,381185	266,0445	899,0174	317,8344	5,618859	1,986465	0	
66.	F	51	16,55	4,222222	76	5,01684	1,75848	2,81874	109,0007	0,97094	85,99616	152	71	34	50,8	21,98753	0,467105	0,223684	8,091889	0,344459	177,9207	574,5241	275,1242	3,779764	1,810028	0	
67.	M	62	6,34	7,5	135	2,66358	0,5172	0,72408	28,00017	3,10475	274,9877	167	108	42	87,4	31,33852	0,646707	0,251497	9,828854	4,287855	308,0217	1061,516	412,8119	6,356385	2,471927	1	

68.	M	62	12,33	4,833333	87	5,6892	3,41352	1,70676	66,00041	1,25319	110,995	173	112	45	91,15	30,45541	0,647399	0,260116	8,482246	0,734251	258,3303	950,0116	381,7011	5,491397	2,206365	1	
69.	F	56	93,58	5,333333	96	3,95658	1,65504	1,37058	53,00033	1,86285	164,9926	169	130	44	110,4	38,65411	0,769231	0,260355	8,977102	1,359169	347,0019	1167,023	394,9925	6,905463	2,337234	1	
70.	F	55	5,54	4,555556	82	6,62016	3,46524	2,87046	111,0007	0,64353	56,99745	166	77	35	64,3	23,3343	0,463855	0,210843	7,756579	0,224191	180,9943	597,2566	271,4803	3,597931	1,635423	0	
71.	M	63	8,51	4,833333	87	4,42206	2,61186	1,37058	53,00033	0,97094	85,99616	177	107	42	92,4	29,49344	0,60452	0,237288	8,227064	0,708415	242,6444	880,2958	345,5367	4,973423	1,952185	0	
72.	M	66	7,85	5,611111	101	4,42206	2,37912	1,75848	68,00042	0,63224	55,9975	180	86	36	78,9	24,35185	0,477778	0,2	7,94728	0,359538	193,531	683,4661	286,1021	3,797034	1,589456	0	
73.	F	41	39,53	4,166667	75	5,12028	2,89632	1,24128	48,0003	2,13381	188,9916	154	99	94	72,5	30,57008	0,642857	0,61039	8,866043	1,71904	271,0356	877,7383	833,4081	5,699599	5,411741	1	
74.	M	67	1,98	4,41	79,38	4,1376	1,83606	1,9395	75,00047	0,7903	69,99687	180	83	39	69	21,2963	0,461111	0,216667	7,92955	0,407476	168,87	658,1526	309,2524	3,656404	1,718069	0	
75.	F	59	14,75	4,5	81	6,10296	3,43938	1,83606	71,00044	1,85156	163,9927	154	84	36	59	24,87772	0,545455	0,233766	8,801124	1,008442	218,9519	739,2944	316,8405	4,800613	2,057406	0	
76.	F	63	26,6	6,166667	111	4,37034	1,99122	0,9051	35,00022	3,26281	288,9871	170	98	40	82	28,3737	0,576471	0,235294	9,682765	3,604917	274,7359	948,911	387,3106	5,581829	2,278298	0	
77.	M	68	8,74	6,555556	118	5,06856	2,53	1,21542	47,00029	4,7418	419,9812	173	102	39	83	27,7323	0,589595	0,225434	10,11775	3,901367	280,5884	1032,01	394,5922	5,965377	2,280879	0	
78.	F	79	18,82	1,777778	32	3,25836	1,44816	1,57746	61,00038	0,54192	47,99785	159	95	38	67,3	26,62078	0,597484	0,238994	6,643745	0,34354	176,8617	631,1558	252,4623	3,969533	1,587813	0	
79.	M	85	7,8	5,444444	98	4,37034	2,56014	1,52574	59,00037	0,58708	51,99768	183	101	39	77,5	23,14193	0,551913	0,213115	7,843019	0,384784	181,5026	792,145	305,8778	4,328661	1,671463	0	
80.	F	68	50,47	5,333333	96	5,06856	2,68944	1,65504	64,0004	1,5806	139,9937	164	99	38	88,5	32,90452	0,603659	0,231707	8,812799	0,955022	289,9809	872,4671	334,8864	5,319921	2,04199	1	
81.	F	60	38,49	9,111111	164	5,5599	2,89	1,08612	42,00026	6,22079	550,9754	165	117	42	107	39,30211	0,709091	0,254545	10,71841	5,727535	421,2561	1254,054	450,1732	7,600327	2,728322	1	
82.	F	67	23,3	5,666667	102	4,37034	2,17224	1,83606	71,00044	0,80159	70,99683	172	99	35	85	28,73175	0,575581	0,203488	8,194461	0,436582	235,4412	811,2516	286,8061	4,716579	1,667477	0	
83.	M	64	4,18	4,777778	86	6,02538	3,4911	1,293	50,00031	2,74347	242,9891	172	94	38	74,5	25,18253	0,546512	0,22093	9,254217	2,121787	233,0446	869,8964	351,6602	5,057537	2,044536	0	
84.	M	42	10,49	4,722222	85	5,48232	9,051	1,13784	44,00027	5,36275	474,9788	178	105	44	93	29,35235	0,589888	0,247191	9,912774	4,713097	290,9633	1040,841	436,1621	5,847423	2,450349	0	
85.	F	33	11,01	4,222222	76	7,34424	4,0083	2,87046	111,0007	1,03868	91,99589	161	80	35	54,5	21,02542	0,496894	0,217391	8,15933	0,361851	171,5534	652,7464	285,5766	4,054325	1,773767	0	
86.	M	56	6,94	4,833333	87	5,14614	2,50842	2,04294	79,00049	1,30964	115,9948	170	102	42	83	28,71972	0,6	0,247059	8,526306	0,641057	244,8732	869,6833	358,1049	5,115784	2,106499	0	
87.	F	58	16,25	4,611111	83	5,87022	3,41352	2,0688	80,0005	0,84675	74,99664	164	107	39	81,5	30,3019	0,652439	0,237805	8,043137	0,409295	243,7224	860,6156	313,6823	5,247656	1,912697	1	
88.	M	66	3,54	4,5	81	4,5255	2,74116	1,00854	39,00024	1,70479	150,9933	172	102	41	82,5	27,8867	0,593023	0,238372	8,718537	1,690354	243,1312	889,2908	357,46	5,170295	2,078256	0	
89.	F	63	13,4	5,111111	92	5,19786	2,56014	2,22396	86,00053	0,91449	80,99638	158	85	36	63	25,23634	0,537975	0,227848	8,223046	0,411199	207,5196	698,9589	296,0297	4,42379	1,873605	0	
90.	F	70	16,68	5,166667	93	6,465	4,06002	2,0688	80,0005	0,76772	67,99696	155	86	34	58,5	24,34964	0,554839	0,219355	8,058915	0,371094	196,2317	693,0667	274,0031	4,471398	1,767762	0	
91.	M	32	5,77	3,944444	71	4,21518	1,91364	1,83606	71,00044	1,03868	91,99589	167	80	39	62	22,23099	0,479042	0,233533	8,091277	0,565711	179,8771	647,3021	315,5598	3,876061	1,88958	0	
92.	F	36	11,06	5,277778	95	4,31862	2,17224	1,34472	52,00032	1,73866	153,9931	166	82	33	60	21,77384	0,493976	0,198795	8,897638	1,292953	193,7358	729,6063	293,622	4,395219	1,768807	0	
93.	M	67	8,53	4,611111	83	4,70652	2,1981	1,8102	70,00043	1,51286	133,994	170	91	46	75,3	26,05536	0,535294	0,270588	8,623489	0,835742	224,6881	784,7375	396,6805	4,616103	2,333415	0	
94.	F	63	28,7	6,333333	114	8,32692	5,58576	2,01708	78,00048	1,59189	140,9937	163	94	35	84	31,61579	0,576687	0,214724	8,991766	0,789205	284,2818	845,226	314,7118	5,185436	1,930747	1	
95.	F	66	9,7	6,833333	123	4,73238	2,09466	2,30154	89,00055	0,71127	62,99718	154	85	29	59,8	25,21504	0,551948	0,188312	8,262127	0,309041	208,3299	702,2808	239,6017	4,560265	1,555855	0	
96.	F	65	106,5	4,888889	88	4,88754	2,74116	1,65504	64,0004	1,10642	97,99562	158	107	35	87,5	35,05047	0,677215	0,221519	8,369112	0,668516	293,3413	895,495	292,9189	5,66769	1,853917	1	
97.	M	64	12,5	5,333333	96	5,92194	3,64626	1,6809	65,0004	1,30964	115,9948	176	103	42	84,4	27,2469	0,585227	0,238636	8,624747	0,77913	234,9976	888,3489	362,2394	5,047437	2,058178	0	
98.	F	78	20,03	4,888889	88	3,7497	1,8102	1,47402	57,00035	1,02739	90,99593	165	100	43	75	27,54821	0,606061	0,260606	8,295004	0,696999	228,5125	829,5004	356,6852	5,027272	2,161728	0	
99.	F	64	34,55	4,833333	87	7,68042	4,03416	3,41352	132,0008	0,54192	47,99785	164	93	35	87	32,34682	0,567073	0,213415	7,643917	0,158757	247,2564	710,8843	267,5371	4,33466	1,631324	1	
100.	F	69	25,92	5,222222	94	3,64626	1,34472	1,88778	73,00045	0,91449	80,99638	158	100	37	72	28,84153	0,632911	0,234177	8,244552	0,484426	237,7855	824,4552	305,0484	5,218071	1,930686	0	
